# The Relationship Between Confidence and Conformity in a Non-routine Counting Task With Young Children: Dedicated to the Memory of Purificación Rodríguez

**DOI:** 10.3389/fpsyg.2021.593509

**Published:** 2021-05-31

**Authors:** Ma Oliva Lago, Ana Escudero, Cristina Dopico

**Affiliations:** ^1^Departamento de Investigación y Psicología en Educación, Facultad de Psicología, Universidad Complutense de Madrid, Madrid, Spain; ^2^Departamento de Investigación y Psicología en Educación, Facultad de Educación, Universidad Complutense de Madrid, Madrid, Spain

**Keywords:** counting, detection task, pseudoerrors, metacognitive monitoring, confidence, conformity, unanimous majority

## Abstract

Counting is a complex cognitive process that is paramount to arithmetical development at school. The improvement of counting skills of children depends on their understanding of the logical and conventional rules involved. While the logical rules are mandatory and related to one-to-one correspondence, stable order, and cardinal principles, conventional rules are optional and associated with social customs. This study contributes to unravel the conceptual understanding of counting rules of children. It explores, with a developmental approach, the performance of children on non-routine counting detection tasks, their confidence in their answers (metacognitive monitoring skills), and their ability to change a wrong answer by deferring to the opinion of a unanimous majority who justified or did not justify their claims. Hundred and forty nine children aged from 5 to 8 years were randomized to one of the experimental conditions of the testimony of teachers: with (*n* = 74) or without justification (*n* = 75). Participants judged the correctness of different types of counting procedures presented by a computerized detection task, such as (a) pseudoerrors that are correct counts where conventional rules are violated (e.g., first counting six footballs, followed by other six basketballs that were interspersed along the row), and (b) compensation errors that are incorrect counts where logical rules were broken twice (e.g., skipping the third element of the row and then labeling the sixth element with two number words, 5 and 6). Afterwards, children rated their confidence in their detection answer with a 5-point scale. Subsequently, they listened to the testimony of the teachers and showed either conformity or non-conformity. The participants considered both compensation errors and pseudoerrors as incorrect counts in the detection task. The analysis of the confidence of children in their responses suggested that they were not sensitive to their incorrect performance. Finally, children tended to conform more often after hearing a justification of the testimony than after hearing only the testimonies of the teachers. It can be concluded that the age range of the evaluated children failed to recognize the optional nature of conventional counting rules and were unaware of their misconceptions. Nevertheless, the reasoned justifications of the testimony, offered by a unanimous majority, promoted considerable improvement in the tendency of the children to revise those misconceptions.

## Introduction

Current approaches assume that counting skills are a foothold in arithmetic performance and mathematical academic achievement during primary school (Chan et al., 2017; Chu et al., 2018; Geary et al., 2018). The majority of preschoolers can repeat the number words and use the standard counting sequence to enumerate a set of objects, but it does not imply that they have a meaningful grasp of counting (Paliwal and Baroody, 2020). In fact, counting is a complex cognitive process where conceptual advance depends on the comprehension of the different properties of logical and conventional counting rules. Exploring the conceptual understanding of counting rules is insufficient in the challenge of laying the foundations for educational intervention programs in the field of mathematics. In other words, in addition to domain-specific knowledge, other cognitive factors, such as metacognitive skills, which also influence school performance of the children, should be explored (Roebers et al., 2012; De Neys et al., 2014; Lubin et al., 2015). Hence, in the current study, we measured the conceptual understanding of counting rules in children from 5 to 8 years of age by means of a counting detection task. Furthermore, to improve the understanding of the failures of children in the detection task, we also measured other two abilities: the confidence in their own judgments (i.e., metacognitive monitoring), and the ability to change a wrong answer by deferring to the opinion of a unanimous majority. These are the issues we will cover in this section.

The logical rules are essential and related to the how-to-count principles posited by Gelman and Gallistel (1978): one-to-one correspondence (every item must be tagged once and only once); stable order (the tags, regardless of their nature, must be ordered in a stable list of unique tags); and the cardinal principle (the last tag used in a count represents the last item and the cardinality of the set). On the contrary, the nature of conventional rules is different. They are optional rules associated with social customs. For example, in cultures where reading and writing go from left to right, counting tends to follow the same spatial organization (Göbel et al., 2018). Conventional rules described in the previous literature include starting from one end of the row, pointing to the elements once, counting adjacent objects consecutively (spatial adjacency), or saying all the number words aloud consecutively (temporal adjacency) (Rodríguez et al., 2013). Initially, conventional rules play a key role because they facilitate the acquisition of the procedure (Briars and Siegler, 1984). However, children need to understand that only the logical rules are relevant to establish the correct solution. For example, counting all elements only once but non-consecutively will lead to a correct count, whereas counting the same element twice, violating the one-to-one correspondence, will result in a wrong answer.

Empirical evidence with the detection paradigm has demonstrated that children have misconceptions about the non-essential nature of conventional counting rules even well into primary school (LeFevre et al., 2006; Kamawar et al., 2010; Rodríguez et al., 2013; Escudero et al., 2015; Lago et al., 2016, 2019). In these studies, children observed a character performing standard correct counts (that comply both with logical and conventional rules), erroneous counts (which break logical rules), and pseudoerrors (not conventional but correct counts that respect logical rules) and were asked to evaluate the correctness of their performance. Overall, the findings showed that, although children successfully detected erroneous counts as incorrect, they did not consider pseudoerrors as correct counts. It has been shown that some characteristics of the task can help children accept pseudoerrors as valid counts. These include the explicit mention of the cardinal value after the count (Lago et al., 2016) or listening to the unanimous opinion of a group of teachers regarding its correctness (Lago et al., 2019). Nevertheless, longitudinal and cross-sectional studies on conventional counting rules agree in stating a slow developing rate during the primary school.

Although pseudoerrors have been broadly used in the study on counting comprehension during the last four decades, there might be some concerns about the possibility that the performance of the children on these trials misrepresents their knowledge of counting. The failure of the children to detect pseudoerrors may not necessarily imply that they do not understand the optional nature of conventional rules. Actually, failures may be due to the existence of different judging criteria, such as judgments basis on what it is typically done: the oddness of the procedure or their likelihood to result in an error, but children still implicitly recognize the acceptability of unconventional procedures. In that sense, asking children to argue their responses would allow to overcome these criticisms, as observed by Rodríguez et al. (2013), Escudero et al. (2015), and Lago et al. (2016). Accordingly, the percentage of kindergarten and primary school children who rejected pseudoerrors as a “risky” or “weird” way of counting was very low (<4.4% of the justifications as studied by Rodríguez et al., 2013).

Evaluating the judgments of children when detecting other kinds of non-routine counting trials could also contribute to improve the understanding of the comprehension of the counting rules of children and its developmental course. Compensation errors seem to be an interesting choice because in contrast to the erroneous counts that have been regularly used in literature, they have superficial similarities to pseudoerrors, even though they are conceptually opposed. Compensation errors occur when the character makes two counting errors, so that the effect of one is canceled out by the second. Consequently, there are several items involved and the last numeral corresponds to the correct cardinal of the set, as in the case of pseudoerrors that only contravene conventional rules. This balances the performance demands of compensation errors and pseudoerrors, and allows establishing more precisely whether children distinguish essential counting aspects, which are governed by logical rules, from non-essential counting aspects, which are governed by conventional rules. This information, together with that given by the justifications of children, will provide a degree of insight into the underlying thought processes. In sum, it could be assumed that the correct performance of children on pseudoerrors and compensation errors requires an explicit reflection on counting rules. One of the novel aspects of the current work consists in presenting kindergarteners and first and second graders with a detection task with these non-routine trials (pseudoerrors and compensation errors) to deepen the misconceptions of the children about conventional rules.

To extend the analysis beyond external factors, such as characteristics of tasks that children face, we address internal factors, such as confidence in their own answers. Confidence judgments, which are considered as a form of metacognitive monitoring, refer to the subjective estimation of the correctness of one's performance in a specific task (Roebers, 2002; Roebers and Howie, 2003; Roebers et al., 2007; Lyons and Ghetti, 2010; Händel et al., 2020; Smortchkova and Shea, 2020). Although the overall level of confidence is high not only in children, but also in individuals of all ages, research on confidence development has consistently shown that children only become able to make accurate confidence judgments about their performance in the middle and late childhood (see, for instance, Lyons and Ghetti, 2010; Roebers et al., 2014; Spiess et al., 2016; van Loon et al., 2017).

However, the overconfidence of children in their performance has not only been viewed as a negative bias, but also proposed as a double-interpretation (Roebers et al., 2007). Roebers et al. considered overconfidence either as a risk factor, for instance, when it represents an obstacle to conceptual change because children continue to stick with their incorrect performance (Smortchkova and Shea, 2020), even in the face of disconfirming evidence (Schneider, 1998), or as a protective factor, for example, when it maintains the motivation that allows children to persevere in the task (Bjorklund and Bering, 2002). De Neys et al. (2014) and Lubin et al. (2015) suggest a different interpretation of overconfidence because they do not conceptualize it as a generalized bias. In their view, if children have an adequate understanding of the concept being measured, they will be less prone to making high and inaccurate confidence judgments. Their studies represent strong evidence that metacognitive development of young children may differ across cognitive functioning and domains (Lyons and Ghetti, 2010; Vo et al., 2014).

De Neys et al. (2014) presented the number conservation task to 5-year-old children. This task assesses understanding of the conservation principle by children, which states that an initial relationship of equivalence between two rows of objects remains the same if only superficial perceptual transformations are performed on the rows (Piaget and Szeminska, 1941). The authors aligned with developmental inhibitory accounts that, contrary to the traditional view, defend that non-conservers might be able to grasp the conservation principle. The failure to pass the task seems to lie in the difficulties of the non-conservers to override the misleading visuospatial intuitions generated by the perceptual transformations. De Neys et al. (2014) examined sensitivity of children in detecting conservation errors, as they sought to establish whether or not the children were aware of the need to inhibit visuospatial impressions. For this purpose, they presented two versions of the conservation task that either include visuospatial cues or do not include them. The former was a conflict task similar to the classical conservation task. The latter was a control task, because it started with two rows of different lengths and the perceptual transformation, quantitatively irrelevant, made the two rows the same length in the final state.

De Neys et al. found that the confidence judgments of the non-conservers reflected their awareness of the fact that their erroneous intuitive answers were questionable. Interestingly, this only occurred in the conflict situation, in which the misleading perceptual cues were not omitted in the end state. Specifically, the authors reported that the lower confidence for conflict vs. non-conflict tasks was only observed in the case of non-conservers. This finding suggests that non-conservers have some understanding of the conservation principle even though they did not inhibit the intuitive responses.

Further, it is also noteworthy to highlight that lower confidence judgments did not favor willingness of children to revise their incorrect responses when a correct answer was presented by means of a Piagetian countersuggestion. Nevertheless, as pointed out in the literature on metacognitive development, younger children do not necessarily use the output of the metacognitive monitoring of their performance to regulate it (i.e., metacognitive control; Lyons and Ghetti, 2010; Vo et al., 2014).

Later, these findings were corroborated by Lubin et al. (2015), who followed the logic of the previous study. Lubin et al. showed conflict and no-conflict comparison word problems to children aged from 8 to 11 years. They found that children whose incorrect answers were based on key words (i.e., the “add if more/subtract if less” heuristic) showed lower confidence judgments than in correctly solved problems. Hence, children noticed that they had solved the problems incorrectly.

The question here is to examine whether children, aged from 5 to 8 years, are also sensitive to their incorrect performance in trials containing pseudoerrors, and whether, as a consequence, they are able to selectively withdraw their previously incorrect responses about conventional counting after listening to the testimony of others.

Recently, the influence of testimony in learning of children has been broadly examined from a developmental point of view (Morgan et al., 2015; Flynn et al., 2018; Harris et al., 2018). It is crucial to know about the determinants of confidence of children in others to guide their learning and behaviors. One of the most striking findings is that children do not follow testimonies blindly. They are selective in deciding which information to endorse, and therefore several factors have already been described. For instance, it is worth mentioning that children defer to the claims of the informants based on (a) their ability to mentally represent the information (Lane et al., 2014), (b) their prior knowledge (Corriveau and Harris, 2010; Chan and Tardif, 2013; Lane et al., 2014; Seston and Kelemen, 2014; Bernard et al., 2015; Enesco et al., 2017; Lago et al., 2019), (c) certain epistemic aspects of the informants (Koening and Harris, 2007; Einav, 2014; Bernard et al., 2015; Rakoczy et al., 2015), (d) whether the testimony is provided by a unanimous rather than a partial majority (Haun et al., 2013; Morgan et al., 2015; Flynn et al., 2018; Lago et al., 2019), or (e) whether the information is consistent or not with their own knowledge or experience (Corriveau and Harris, 2010; Enesco et al., 2017; Lago et al., 2019).

In particular, learning how to count is usually conducted in a testimonial context. People children trust (e.g., parents or teachers) give them information about the way to do it. Children repeat and practice the procedure taught, but, as it has been previously stated, they are still far from understanding the rationale behind it. In this regard, some researchers have addressed whether tendency of the children to conform allows them to question their own erroneous conception of conventional counting rules by using the testimony given by a qualified majority of math teachers (Enesco et al., 2017; Lago et al., 2019).

Participants who were studied by Enesco et al. (2017) faced two conflicting perspectives: correct claims that run counter to their own knowledge (because the majority of teachers or one dissenter accepted as correct the counting pseudoerrors), and erroneous claims (where teachers judge pseudoerrors as incorrect counts, similar to own judgments of children). They found that both kindergartners and second graders endorsed claims that considered pseudoerrors as incorrect counts, irrespective of the source of information (majority or dissenter). The authors concluded that children weighted the arguments of the informants and sided with claims that were not counterintuitive to them.

Lago et al. (2019) extended these findings by modifying the experimental situation, testing conformity following an Asch-style paradigm. Children aged from 5 to 7 years faced only correct majority claims (accepting pseudoerrors as valid counts) that run counter to their own knowledge. The majority could be unanimous (four teachers in agreement) or not (three teachers vs. one dissenter). The tendency of children to conform in the unanimous condition was five times greater than in the non-unanimous one, regardless of their age. Besides, this effect was maintained over time in the primary school children.

To summarize, these two studies suggest that only the unanimous majority scenario encouraged children to re-examine their own misconceptions about conventional counting rules. It can be concluded that this unanimous scenario promotes informational (informative influence, as defined by Deutsch and Gerard, 1955) over social motivation to accept the testimony. If normative influence (social pressure) were responsible for deference of children to opinions of the teachers, they would have accepted the majority testimony even in the non-unanimous conditions. However, this hypothesis needs further evidence. Thus, the current study presents an extension to the existing understanding of the relationship between the majority testimony and greater acceptance of pseudoerror trials by children.

The aim of the current research was three-fold; first, to analyze competence of children to distinguish logical from conventional counting rules using two non-routine counting strategies. Both yield correct cardinal values, but violate either logical (i.e., compensation errors) or conventional (i.e., pseudoerrors) counting rules. To do this, we used an error-detection paradigm to place fewer performance demands on the children, keep task demands constant, and present unfamiliar counting stimuli. Given that children had rarely seen counting strategies like those performed by the characters, they would detect them correctly if they focused on the conceptual and abstract aspects of counting rules, rather than on the superficial aspects of counting performance. Consistent with previous literature, if children find it easier to recognize the essential nature of logical counting rules than the optional nature of the conventional ones, participants would perform better on the compensation errors than on the pseudoerrors. Also in accordance with the above-mentioned research, we expected a slow developmental pace of ability of children to distinguish logical from conventional counting rules.

The second goal was to examine confidence of children in their own judgments about performance of other to explore potential age differences in sensitivity of children to their inaccurate answers. To this end, on the one hand, we assessed whether the nature of the non-routine counts of the detection task has any influence on confidence ratings of the children. We expected that children with more developed metacognitive skills would show less confidence in their responses to pseudoerrors than to compensation errors, since the arbitrariness of conventional counting rules seems to be less evident than the essential nature of logical rules. On the other hand, we were also interested in determining whether confidence ratings of children changed with age. The older the children are, the more learning experience they have, which enhances their understanding of the meaning of counting. In accordance with the previous research findings about pronounced overconfidence bias of young children with regard to what they know, and the potential metacognitive monitoring differences across several forms of cognitive functioning and domains, we expected a slow development in the age range considered here.

The third aim was to further examine the relationship between the testimony of the majority and children's increased acceptance of pseudoerrors as valid counts. We tested the assumption that children respond by informational influence with an Asch style paradigm that included two different experimental conditions: listening to the testimony of an unanimous majority with articulated arguments (with justification) or not (without justification). Putting it differently, would a unanimous majority that offered no arguments still have the same influence on conformity of children as a unaminous majority that did offer arguments? According to the informative influence hypothesis, we expected that children, regardless of their age group, would be more likely to conform when the unanimous arguments of the majority were articulated than when they were not, since they weighted the claims of the informant. On the contrary, if children respond based on the normative influence (or social motivation) instead, the presence of articulated arguments would not have any effect on tendency of the children to conform. Furthermore, we also analyzed whether metacognitive monitoring skills of children qualified this relationship between the testimony of the majority and acceptance of pseudoerror trials of children and whether it changed across the age-level. It could be expected that children who noticed their incorrect performance would be more able to conform to the unanimous majority.

## Materials and Methods

### Participants

A total of 149 children (75 girls, 50.3%) took part in the study. They came from either a private (*n* = 47) or a semi-private (*n* = 102) school in the Madrid area. All participants were Spanish speakers, without learning difficulties, predominantly Caucasian and from middle- or middle/high-class families. All parents gave their written informed consent for the study and children took part voluntarily. The Deontological Commission of Psychology, Complutense University of Madrid, approved the study.

The kindergarten group consisted of 47 children (21 girls, 44.7%) with a mean age of 69.7 months [standard deviation (*SD*) = 4.05]; the first-grade group, 51 children (29 girls, 56.9%) with a mean age of 71.4 months (*SD* = 7.4); and the second-grade group, 51 children (25 girls, 49%) with a mean age of 82.2 months (*SD* = 6.9). All participants were randomized to one of the experimental conditions of testimony of the teachers: with or without justification. In the latter, there were 75 participants: 23 kindergartners, 26 first graders, and 26 second graders. In the former, there were 74 participants: 24 kindergartners, 25 first graders, and 25 second graders (see Table 1).

**Table 1:** Sample characteristics as a function of each experimental condition of the testimony of teachers.

**Characteristics**	**Testimony without justification**			**Testimony with justification**		
	**Kindergarten**	**1st grade**	**2nd grade**	**Kindergarten**	**1st grade**	**2nd grade**
*N*	23	26	26	24	25	25
Gender Girls Boys	12 11	15 11	14 12	9 15	14 11	11 14
*M* age in months (*SD* in months)	69.4 (4.7)	73.8 (8.1)	82.3 (6.9)	70 (3.4)	68.9 (5.6)	82.2 (7.1)

### Materials and Procedure

Participants were tested individually, in a single session, in a quiet room near their own classroom. A female experimenter conducted the semi-structured interview that lasted about 20–25 min.

**Figure 1:**
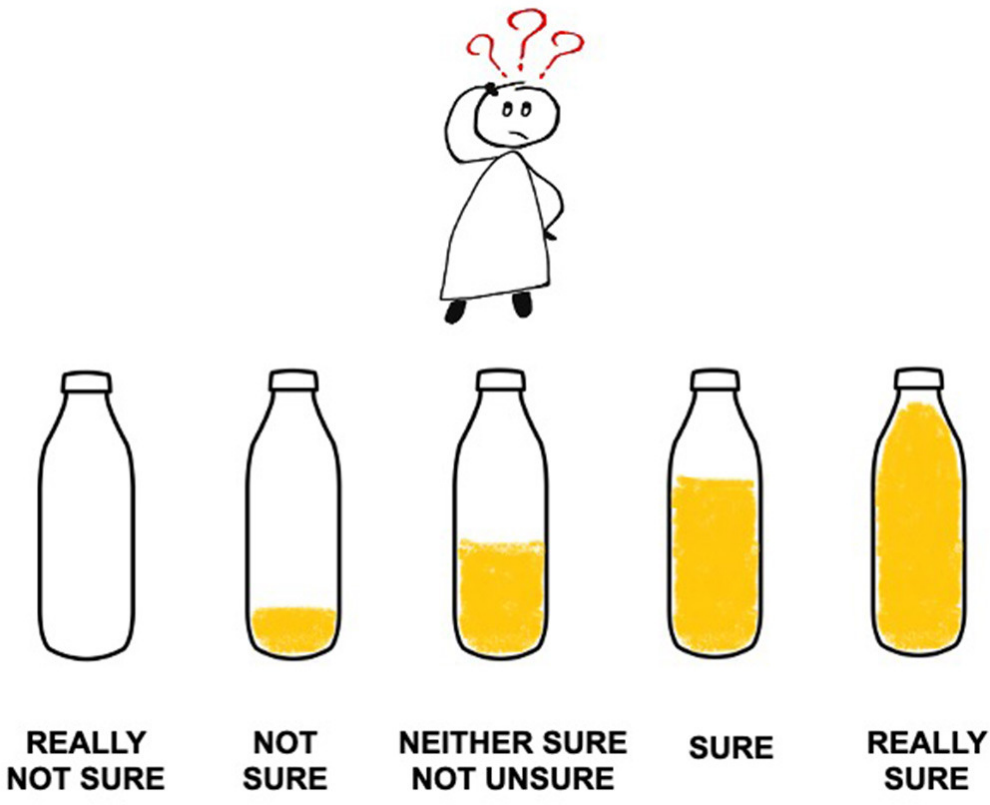
Five-point confidence scale. The drawings used in this figure are available as a free download in the FreeIMG.net portal.

Before starting the experiment, the researcher explained to the participants that they were going to see some cartoon girls counting. Afterward, children were instructed in the use of the 5-point scale to rate their confidence judgments. It was printed on a separate sheet of paper and consisted of five different bottles, representing each level of the scale, from an empty bottle (“really not sure”) to a totally full bottle (“really sure”) (Figure 1). According to the research literature, pictorial scales work well to assess the metacognitive monitoring in young children (Destan et al., 2014; Lubin et al., 2015). Additionally, as the use of smileys in the confidence scales seems to induce the selection of the happy faces—which represent “very sure” responses—by social desirability (Roebers and Howie, 2003), more neutral elements, such as bottles, have been employed.

We drew on the works of De Neys et al. (2014) and Lubin et al. (2015) to devise the procedure. Thus, we explained each item on the scale and asked some practice questions in order to ensure the children's understanding of it. Two of the practice questions were designed to be very simple (e.g., “Are zebras blue?”) and the other two were intended to be very hard (e.g., “How many fingers am I sticking under the table right now?”). In the latter, the experimenter explicitly alluded to the difficulty of the questions, pointing out that it was usual to feel unsure about the correctness of the responses.

Once the researcher checked the understanding of the rating scale of the children for confidence judgments, the three-phase counting task began. Participants completed consecutively all the phases for each counting trial (i.e., detection task, rating their confidence in their detection answer, and listening to testimony of the teachers) before starting a new one. All the interviews were recorded in audio and the responses of the children throughout the task were subsequently transcribed for data analyses.

#### Counting Detection

Children were shown a video of a computerized counting detection task^1^ where four cartoon girls counted different arrays of items. The video started with Rosa, one of the characters, presenting the game: “*We are going to play counting things with some girls. I am going to put some things on the table for them to count. You must pay attention and tell me if they have done it right or wrong*.” Next, one of the four cartoon girls counted the objects that appeared on the scene aloud as she touched them (then, the object made a slight movement as a sign of being counted). The objects were always presented in a row, the set size ranging from seven to 12 items, and the instructions were the same for the eight trials presented. When the character finished the count, the participants were asked “*Has she done it right or has she done it wrong?*” and immediately afterward they always had to justify their answers (i.e., “*Why?*” or equivalent expressions depending on the answers of children, for instance, “*Why do you think that?*”).

The eight counting trials presented were of two types: routine or familiar and non-routine or non-familiar. The two routine trials include a correct conventional count and an erroneous count that violated the logical rule of one-to-one correspondence, because the character split the Spanish word “seven” into two tags (“se – ven”) to count two different objects (the seventh and the eighth). As previous research has shown that these kinds of trials are detected easily by the children of the age range evaluated here (Muldoon et al., 2003; LeFevre et al., 2006; Kamawar et al., 2010; Rodríguez et al., 2013; Escudero et al., 2015; Lago et al., 2019), they have been included as control trials and were excluded from the analysis.

The non-routine trials, which are conceptually more demanding than the routine ones, were comprised of six different ones: three pseudoerrors and three compensation errors (see Table 2 for a description). Two of the three different compensation errors were created for the current study. Compensation error 1 was presented to a small group of 4- and 5-year old children by Gelman and Meck (1986).

**Table 2:** Description of the non-routine trials.

**Trials**	**Set size**	**Trial description**
**Transgressions of spatial rules**		
Pseudoerror 1	12	Counting first six footballs, followed by other six basketballs that were interspersed along the row.
Compensation error 1	8	Skipping the third item and then double-counting the six elements with the tags “5” and “6”.
**Transgressions of temporal rules**		
Pseudoerror 2	8	Pointing to all elements counted out loud the first five items of the row, counting the sixth and the seventh silently, and the eighth out loud again.
Compensation error 2	9	Tagging the second element “3” (instead of “2”) and repeating the tag “7” for both the sixth and the seventh elements.
**Transgressions of temporal-spatial rules**		
Pseudoerror 3	10	Pointing to and tagging three consecutive times with the same tag the sixth element (“6,6,6”).
Compensation error 3	8	Skipping the number word “3”, tagging the third element as “4”, (temporal violation) and skipping the sixth item (spatial violation).

There were three different orders of presentation of the trials in which the correct conventional and the erroneous counts had a fixed position. Order 1: Ps 1, CE 2, correct conventional, Ps 3, CE 1, erroneous count, Ps 2 and CE 3; order 2: Ps 2, CE 3, correct conventional, CE 1, Ps 1, erroneous count, Ps 3 and CE 2; order 3: Ps 3, CE 3, correct conventional, Ps 2, CE 2, erroneous count, Ps 1 and CE 1. Children were randomized to one of these three orders.

#### Confidence Judgments

Immediately after responding to the detection task, participants reported whether they were sure or unsure about their answers: “*Which bottle do you choose to tell me how sure you are that _____* [the name of the cartoon character] *has done it _____* [right/wrong]*?*” As previously stated, confidence judgments were made by means of the 5-point Likert scale depicted in Figure 1 and at a local level. In other words, they were item-specific in order to measure the monitoring abilities of the children in each kind of the counting trial. To avoid lengthy test sessions and prevent children from fatigue, confidence judgments were only asked in five of the eight counting trials, randomly selected.

#### Influence of Testimony on Decisions of Children

During the last part of the task, participants watched a video with the testimony of the teachers about the last counting trial. The experimenter introduced the task saying: “*Now, you are going to see some teachers. All of them are math teachers. They have also watched* _____ [the name of the cartoon character] *counting and we have asked them whether*_____ [the name of the cartoon character] *has done it right or wrong. Listen carefully*.” The four teachers were female real-life adults and always gave a unanimous correct response (i.e., considering the correct conventional and the pseudoerrors as correct counts; and the erroneous count and the compensation errors as incorrect ones). Before emitting the final response, teachers were shown sharing their thoughts, although only one of them expressed their opinion aloud by saying “*We all think that…*.” The representative (spokesperson) and the teachers' spatial location changed from trial to trial.

In the “without justification” condition, the teachers only stated whether the counting strategy of the character was right or wrong. In the “with justification” condition, they also reasoned their answers. Their arguments were accessible to children, since they were based on those given by other children in previous studies (Lago et al., 2019). The specific justifications stated by the teacher are detailed in Table 3. After the responses from the teachers, the experimenter reminded the participants to make sure they understood the situation: “*All the teachers said that… Are math teachers right or not?*” In addition, the participants were always asked to justify their decision.

**Table 3:** Description of the arguments offered by the teachers in the “with justification” condition in pseudoerrors and compensation errors.

**Rules broken**	**Pseudoerrors**	**Compensation errors**
Spatial adjacency	She counted right, because although she counted the first footballs and then basketballs, she has counted everything.	She counted wrong, because she has not counted one sweet and she has double-counted another sweet.
Temporal adjacency	She counted right. Although she has not said some turtles aloud, she has counted them in her mind.	She counted wrong, because she has skipped a number and then, she has repeated another number.
Temporal-spatial adjacency	She counted right, because it does not matter counting “6” three times in the same bird.	She counted wrong, because she has omitted a number and then, she has skipped a sock.

### Measures

#### Performance on Counting Detection Task

As expected, performance of children in routine trials (correct conventional and erroneous, included as control trials) was quite high: 100% in correct conventional and 97.9% in erroneous counts; 100% in correct conventional and 98% in erroneous counts, and 98% in both trials for kindergarteners, first and second graders, respectively.

The relevant trials in the current study were non-routine. Responses of children to pseudoerrors in the detection task were rated as correct (i.e., scored 1 point) when children “accepted” the counting of cartoon girl as valid counts: they were considered “right” and justified as correct. Otherwise, they scored 0, for example, when children “rejected” the pseudoerrors by judging them as incorrect counts. In the same way, answers of the children to compensation errors were coded by 1 only when they “rejected” them: they judged the trials as “wrong,” alluding to both violations of logical rules. Any other response was scored 0.

As for the justifications, we have employed the categorizations used in previous studies for pseudoerrors and have defined a new one based on the similar criteria for compensation errors (Rodríguez et al., 2013; Lago et al., 2016, 2019). An independent intercoder agreement for 31.6% of the answers was 94.4%. Disagreements were discussed until consensus was reached.

#### Metacognitive Monitoring Judgments

Children could rate their confidence with the 5-point Likert scale (Figure 1). Their scores ranged from 1 (“really not sure”) to 5 (“really sure”). Following Schraw (2009), three different measures of metacognitive monitoring were calculated for confidence judgments: bias, absolute accuracy, and discrimination. To compute bias and absolute accuracy indices, scores of the rating scales were recoded ranging from a maximum of 1 (really sure) to a minimum of 0.2 (really not sure), with intervals at 0.2.

The first measure was the bias index^2^. According to Schraw (2009), the first measure informs about the degree to which a child is underconfident or overconfident. The bias index ranges between −1 (underconfidence) to 1 (overconfidence). The distance from 0 gives information about the gravity of the judgment error. The bias index was calculated for the non-routine trials, pseudoerrors, and compensation errors, separately.

The second measure was taken as the absolute accuracy index^3^. Following Schraw (2009) definition, the second measure indicates how precise the confidence judgment is by calculating the discrepancy between the confidence rating and the real performance in a counting trial. The absolute accuracy index between 0 and 1. The scores closer to 0 revealed small discrepancies, which meant accurate monitoring, whereas scores closer to 1 reflected inaccurate monitoring. As before, the absolute accuracy was also computed for the non-routine trials, compensation errors, and pseudoerrors, separately.

The only difference between these two measures is that the discrepancy between confidence and performance is either squared or not. Absolute accuracy provides information about the metacognitive monitoring accuracy given a specific perfomance, whereas the bias index offers complementary information about the direction of the judgment error. Absolute accuracy and bias index fit with the aims of the current study since they can be calculated for pseudoerrors and compensations errors separately.

Finally, the discrimination index^4^ measures the ability to distinguish between the incorrect and correct performances when emitting confidence judgments. Positive values indicate that children have more confidence about the judgments given in the correct answers than about the ratings given in the incorrect ones. Negative values denote more confidence about their incorrect answers, which could be considered indicative of low metacognitive awareness. The discrimination index was calculated considering the confidence scores in the six non-routine trials altogether.

#### Conformity to Testimony of Teachers

In order to examine whether the tendency of the children to conform allows them to correct their inaccurate responses in the detection task phase, answers of participants endorsing claims of teachers were coded 1 (presence of conformity), and the rest of the answers were coded 0 (absence of conformity). Note that the presence of conformity was only possible when children had failed to detect the trials in the first counting detection task. Due to the high success rate in the detection of compensation errors (Table 4), conformity was exclusively analyzed in pseudoerrors.

**Table 4:** Means and standard deviations (*SD*) of correct responses in the detection task.

**Grade level**	**Compensation errors *M* (*SD*)**	**Pseudoerrors *M* (*SD*)**
Kindergarten	2.09 (0.95)	0.91 (0.72)
First grade	1.98 (0.91)	0.75 (0.66)
Second grade	2.45 (0.73)	1.06 (0.79)

*Maximum score is 3*.

The procedure followed to categorize justifications of children after listening to testimony of the teachers was the same as the one described in the counting detection task, because children scored 1 point only when they accepted claims of teachers and duly justified their conformity.

## Results

Preliminary analyses showed that performance of the children was above the chance level on compensation errors (*M* = 2.17, *SD* = 0.88) and below the chance level on pseudoerrors (*M* = 0.91, *SD* = 0.73): compensation errors, *t*_(148)_ = 9.32, *p* < 0.001, *d* = 0.76, 95% CI [0.53, 0,82]; pseudoerrors: *t*_(148)_ = −9.94, *p* < 0.001, *d* = 0.81, 95% CI [−0.71, −0.48]. Furthermore, a series of one-way ANOVAs were conducted to check whether the order in which the trials were presented affected the performance of the children on the detection task as well as on their confidence in their answers. There were no differences across the three presentation orders neither in the performance of the children nor in their monitoring ability: compensation errors detection, *F*_(2, 146)_ = 0.61, *p* = 0.548, $\eta_{\text{p}}^{2}$ = 0.01; pseudoerrors detection *F*_(2, 146)_ = 0.52, *p* = 0.595, $\eta_{\text{p}}^{2}$ = 0.01; compensation errors absolute accuracy [*F*_(2, 146)_ = 1.80, *p* = 0.169, $\eta_{\text{p}}^{2}$ = 0.02]; pseudoerrors absolute accuracy, [*F*_(2, 146)_ = 2.47, *p* = 0.089, $\eta_{\text{p}}^{2}$ = 0.03]; compensation errors bias [*F*_(2, 146)_ = 1.33, *p* = 0.269, $\eta_{\text{p}}^{2}$ = 0.018]; pseudoerrors bias, *F*_(2, 146)_ = 2.70, *p* = 0.071, $\eta_{\text{p}}^{2}$ = 0.036; and discrimination, *F*_(2, 146)_ = 1.594, *p* = 0.207, $\eta_{\text{p}}^{2}$ = 0.02. Thus, the order of presentation of the trials was excluded from the data analysis.

The first aim of the study is to analyze competence of the children to distinguish logical from conventional rules. Therefore, a mixed ANOVA was performed on the number of correct responses on the detection task. To address the second goal, assessing confidence of the children in their answers to the detection task, more ANOVAs on each one of the three different measures of metacognitive monitoring (i.e., bias, absolute accuracy, and discrimination) were conducted. Finally, a series of binary logistic regression analyses were implemented to determine the probability that children would conform to correct testimonies of teachers in pseudoerrors.

### Counting Detection Task

The mixed analysis of variance 3 (Grade Level: kindergarten, first grade, or second grade of primary education) × 2 (Detection Task: compensation errors or pseudoerrors) with the latter as the repeated measure and conducted on the number of correct responses, revealed a main effect of Grade Level, *F*_(2, 146)_ = 6.97, *p* = 0.001, $\eta_{\text{p}}^{2}$ = 0.09, and detection task, *F*_(1, 146)_ = 177.34, *p* < 0.001, $\eta_{\text{p}}^{2}$ = 0.54 (Table 4). No significant interactions were found.

With regard to the grade level factor, the Bonferroni *post-hoc* tests showed that the second graders obtained significantly better results than the first graders (*p* = 0.001), because they responded correctly in 58.5% of the trials in comparison with the 45.4% of the trials correctly answered by the first graders.

In terms of the detection task factor, children performed significantly better with compensation errors (72.4% of the trials responded correctly) than with the pseudoerrors (30.2% of the trials responded correctly). These two non-routine detection tasks, where either logical or conventional rules were violated, involved counting strategies that led to correct cardinal values. Specifically, the difference between the compensation errors and pseudoerrors was in the counting strategy, that is, in the counting rules that each one of them violated. Therefore, this result indicates that children show a better understanding of the essential nature of the logical than that of the optional character of the conventional rules.

The justifications given by the children revealed that they were able to remember the counting strategy of the characters and that the main reason to reject the compensation errors and pseudoerrors as incorrect counts was the violation of logical rules and conventional rules, respectively. As can be seen in Table 5, the success achieved by the children in the compensation errors showed that the majority were able to reject them as invalid counts and justify their response by alluding to the two logical errors. For example, a second grader rejected the spatial compensation error by saying, “Right but wrong, she skipped one and repeated another. She guessed the right number.” Incorrect responses in the compensation errors occurred because children only mentioned the first error (e.g., a girl from the first grade who rejected the temporal-spatial compensation error gave the following justification, “because she skipped the 3 and instead of getting 8, she got less than 8, because she had counted wrong”) and, to a lesser extent, the second one to justify their rejection judgments.

**Table 5:** Percentages of justifications of children in the detection task.

**Detection Task**	**Kinder garteners**	**First graders**	**Second graders**
**Compensation errors**			
Rejected and correct justification (two logical errors)	69.5	66	81.7
Rejected but only for the first error	19.9	19.6	11.1
Rejected but only for the second error	7.1	5.2	2
Others	3.5	9.2	5.2
**Pseudoerrors**			
Accepted and correct justification	29.8	24.8	35.3
Rejected by violation of conventional rules	67.4	73.8	64
Others	2.8	1.4	0.7

The number of responses in which children accepted the compensation errors as valid counts was very scarce: 2.8%, 5.2%, and 2%, in kindergarten, the first grade and the second grade, respectively. Only three children from second grade accepted them because the two logical errors canceled each other (2% of responses). For example, in the spatial compensation error, “Yes, she did right because she did 1, 2, 3, 4, 5, 6, 7, and 8 and although she skipped this one—pointing out the third element—she has counted this one twice—pointing to the sixth element.” Due to the low frequency of this kind of arguments, they have been embedded in the “others” category.

As for pseudoerrors, children justified their correct answers by explaining that the violation of the conventional rules did not alter the logic of the count (e.g., a second grader boy stated, “She counted right, because she has counted in the same bird 6”). When they judged pseudoerrors as incorrect counts, most of the justifications referred to the violation of different conventional rules, which shows the great importance they give to following these conventional rules. Some examples of the justifications of the children include spatial adjacency [e.g., a boy from the second grade explained that pseudoerror 1 was wrong because “She guessed the number (referring to the cardinal number), but she skipped a few and then the other way around”]; temporal adjacency (e.g., a first grader boy rejected the pseudoerror 2 “Because she skipped two turtles, because in those two she didn't say anything”); and temporal-spatial adjacency (e.g., a girl from the second grade rejected pseudoerror 3 “Because she has repeated the number a lot of times in the same bird”). Table 5 displays the percentages of the different justifications given by children.

### Confidence Judgments

A 3 (Grade Level: kindergarten, first grade, or second grade of primary education) × 2 (Detection Task: compensation errors or pseudoerrors) mixed ANOVA, with the detection task as the repeated measure, was conducted on the bias scores (Table 6). The analysis revealed a main effect in terms of Grade Level, *F*_(2, 146)_ = 4.84, *p* = 0.009, $\eta_{\text{p}}^{2}$ = 0.06, and Detection Task, *F*_(1, 146)_ = 142.59, *p* < 0.001, $\eta_{\text{p}}^{2}$ = 0.49. No significant interactions were found.

**Table 6:** Means and *SD* of bias scores.

**Grade level**	**Compensation errors *M* (*SD*)**	**Pseudoerrors *M* (*SD*)**
Kindergarteners	0.23 (0.36)	0.63 (0.30)
First graders	0.30 (0.38)	0.73 (0.29)
Second graders	0.11 (0.28)	0.63 (0.32)

*Score range goes from −1 to 1*.

As expected, most children were overconfident when they made confidence judgments about their own detection performance, that is, confidence was higher than performance (0.43, 0.52, and 0.37 in kindergarten, the first and the second grade, respectively). However, Bonferroni *post-hoc* tests only revealed significant differences between the first and second graders (*p* = 0.007), indicating that there is not a clear development pattern in our data because children were not progressively less overconfident.

Bias scores also differed depending on the detection task because children provided more overconfident judgments and achieved higher bias scores in pseudoerrors than in the compensation errors (0.66 vs. 0.21, respectively).

Regarding the precision of judgments, the absolute accuracy scores of children were analyzed with a 3 (Grade level: kindergarten, first grade, or second grade of primary education) × 2 (Detection task: compensation errors, or pseudoerrors) mixed ANOVA, with detection task as the repeated measure. There were significant main effects of grade level, *F*_(2, 146)_ = 4.51, *p* = 0.013, $\eta_{\text{p}}^{2}$ = 0.06, and detection task, *F*_(1, 146)_ = 129.14, *p* < 0.001, $\eta_{\text{p}}^{2}$ = 0.47 (Table 7).

**Table 7:** Means and *SD* of absolute accuracy scores.

**Grade level**	**Compensation errors *M* (*SD*)**	**Pseudoerrors *M* (*SD*)**
Kindergarten	0.29 (0.33)	0.65 (0.29)
First grade	0.38 (0.33)	0.75 (0.26)
Second grade	0.23 (0.24)	0.71 (0.24)

*Score range goes from 0 to 1*.

The Bonferroni *post-hoc* tests indicated that there were significant differences between the first graders and the kindergartners (*p* = 0.041), and between the first graders and the second graders (*p* = 0.025). Kindergartners and the second graders registered lower absolute accuracy scores (0.47 in both cases) than the first graders (0.57), suggesting a better monitoring accuracy in those two grade levels.

As for the detection task factor, the children made more accurate confidence judgments in compensation errors (0.3) than in pseudoerrors (0.7), pointing to a better monitoring accuracy in the former.

Finally, with regard to how well-children were able to discriminate between confidence judgments for accurate and inaccurate performance considering grade level, a one-way ANOVA was conducted. Due to the assumption of homogeneity of variance not being met according to the Levene's test (*p* = 0.020), the Welsch's adjusted F ratio was employed, with an alpha level of 0.05. Discrimination index differed across grade levels: Welch's *F*_(2, 92.57)_ = 5.30, *p* = 0.007, and ω^2^ = 0.05. The Games-Howell *post-hoc* tests revealed significant differences between the first and second graders (*p* = 0.004), whose discrimination index means were −0.72 (*SD* = 2.01) and 0.43 (*SD* = 1.51), respectively. This may be the result of the negative scores in the first graders compared to the positive scores of the second graders. The first graders were more confident about incorrect responses, as evidenced in their negative discrimination scores that reflected a difficulty to distinguish between the correct and incorrect responses. On the contrary, positive discrimination scores of kindergarteners (*M* = 0.13, *SD* = 2.32) and positive discrimination scores of second graders can be interpreted as a better metacognitive awareness of the correct performance, because they assigned higher confidence to the correct than to the incorrect counts. However, taking into account the magnitude of the discrimination index and the fact that the maximum score is 5, children barely distinguish between the correct and incorrect performance.

### Influence of Testimony on Decisions of Children

Conformity implies that children accept contrary information provided by others, which makes data on compensation errors inadequate to assess the conformity because the majority of the children, in the same vein as teachers, rejected them as correct counting strategies (Table 5). Therefore, this analysis focused on conformity of children to testimony of the teachers for pseudoerrors and, more specifically, on a dichotomous variable [presence (= 1) or absence (= 0) of conformity] for an individual child. Binary logistic regression was implemented to determine the probability that children would conform to correct testimonies of the teachers. Each pseudoerror was modeled separately, and condition (with justification, without justification), grade level (kindergarteners, the first graders, and the second graders), and absolute accuracy, were included as predictor variables (Table 8). Other metacognitive monitoring rates were excluded to avoid multicollinearity^5^. There were two categorical predictor variables: Grade level and condition. For the former, two dummy variables were created and the second grade became the reference category. As for the latter, the reference category was the without justification level.

**Table 8:** Estimated coefficients, odds ratio (OR), and statistics for predictors in binary logistic regression models.

**Predictor**	**β (*SE*)**	**Odds ratio**	**95% CI**	**Wald**	***df***	***p***
**Pseudoerror 1. Spatial adjacency**						
Constant	−4.21 (1.65)	0.02	–	6.50	1	0.011
Condition	2.54 (0.75)	12.67	2.93–54.77	11.56	1	0.001
Grade level—Kindergarten	0.86 (0.84)	2.37	0.46–12.25	1.06	1	0.304
Grade level—First grade	0.18 (0.82)	1.20	0.24–5.92	0.05	1	0.826
Absolute accuracy	1.27 (1.54)	3.55	0.17–72.96	0.67	1	0.412
**Pseudoerror 2. Temporal adjacency**						
Constant	−1.55 (1.11)	0.21	–	1.95	1	0.162
Condition	2.64 (0.66)	14.01	3.88–50.57	16.25	1	<0.001
Grade level—Kindergarten	0.04 (0.60)	1.04	0.32–3.36	0.004	1	0.947
Grade level—First grade	−0.30 (0.58)	0.75	0.24–2.33	0.26	1	0.613
Absolute accuracy	−1.68 (0.99)	0.19	0.03–1.30	2.88	1	0.090
**Pseudoerror 3. Spatial-temporal adjacency**						
Constant	−0.88 (0.97)	0.42	–	0.82	1	0.365
Condition	1.99 (0.51)	7.35	2.71–19.90	15.40	1	<0.001
Grade level—Kindergarten	0.02 (0.58)	1.02	0.33–3.14	0.001	1	0.980
Grade level—First grade	−0.48 (0.54)	0.62	0.21–1,80	0.78	1	0.378
Absolute accuracy	−1.61 (0.90)	0.20	0.04–1.16	3.21	1	0.073

*β, estimated coefficients for each predictor; SE, standard errors; CI, confidence interval for odds ratio*.

*In categorical predictors, reference categories were “without justification” for condition, and “second grade” for grade level (which was entered as two dummy variables)*.

The preliminary univariate analysis indicated that there were no differences between conditions (with or without justification) in the number of children who accepted (or not) each pseudoerror before listening to testimony of the teachers. This occurred regardless of whether the three age groups were analyzed together or separately.

Data on 65, 121, and 128 children were used to perform the analysis of pseudoerrors 1, 2, and 3, respectively, because the dependent variable was the number of responses in which children failed to consider a pseudoerror as a correct count in the detection task but then accepted it as a valid count after hearing the claims of the teachers. The classification cutoff was set to 0.41 in order to improve the probability of detecting true events of conformity. The model included all the predictor variables entered simultaneously (see Table 8). The predictive power of this model was significantly better than that of the baseline model for the three pseudoerrors: $\chi_{\left(4,\text{N}=65\right)}^{2}$ = 17.44, *p* = 0.002, Nagelkerke's *R*^2^ = 0.034; $\chi_{\left(4,\text{N}=121\right)}^{2}$ = 27.52, *p* < 0.001, Nagelkerke's *R*^2^ = 0.31; $\chi_{\left(4,\text{N}=128\right)}^{2}$ = 22.96, *p* < 0.001, Nagelkerke's *R*^2^ = 0.24, for pseudoerrors 1, 2, and 3, respectively.

The binary logistic regression for pseudoerror 1 showed that condition was the unique significant predictor of conformity of children to testimony of the teachers at the 1% level (Wald = 11.56, *p* = 0.001), after controlling for absolute accuracy and grade level. The odds' ratio (OR) indicated that the odds that children change their response after listening to testimony of the teachers were 12.67 higher for children in the condition with justification than in that without justification. The model correctly classified 79.2% of cases where there was no conformity and 70.6%, where there was conformity, giving an overall correct percentage prediction rate of 76.9%. The model predicted that 15.97% of the children in the condition of testimony with justification would conform whereas only 1.96% of children did so in that without justification.

The results for pseudoerror 2 indicated that condition was the unique significant predictor of conformity of children to claims of teachers at the 1% level (Wald = 16.25, *p* < 0.001), having allowed for absolute accuracy and grade level. The OR revealed that children were 14.01 times more likely to change their judgment after hearing a justification of opinion of the teachers than when no justification was given. The model correctly predicted 85.9% of cases where there was no conformity and 51.7% where there was conformity, with an overall correct prediction rate percentage of 77.7%. The model predicted that 74.81 and 17.36% of the children would conform to the testimony with justification and without it, respectively.

Finally, the binary logistic regression for pseudoerror 3 also revealed that condition was the unique significant predictor of conformity of children to opinion of teachers at the 1% level (Wald = 15.40, *p* < 0.001), after controlling for absolute accuracy and grade level. The OR for condition showed that children in the condition with justification were 7.35 times more likely to conform than children in that without justification. The model correctly classified 86.5% of cases where there was no conformity and 50% were there was conformity, giving an overall correct percentage prediction rate of 77.3%. The model predicted that there would be a higher tendency to conform to opinion of the teachers in the condition with justification (75.19% of the children) than in that without justification (29.58% of the children).

To summarize, and in line with the informational influence hypothesis, children tended to conform more often after hearing a justification of the testimony than after hearing only the testimony of the teachers, regardless of age or kind of the pseudoerror. Based on the third goal of the study, we have found that a unanimous majority that do not provide arguments do not have the same influence on conformity of children, because the children not only considered the content of the testimony but also the justification supporting it.

## Discussion

Decades of intensive research support the view that counting is paramount to arithmetical development at school. It is no longer thought of as an enumeration skill but rather as a complex cognitive process. In the current study, we used two non-routine detection tasks, which differ in the conceptual aspects of counting but resemble each other superficially, in an effort to disentangle the contribution of logical and conventional rules to performance of the children on detection tasks. To further contribute to unravel the nature of failures of children in these non-routine detection tasks, we also measured their metacognitive monitoring abilities and explored the relationship between a majority testimony (articulated or not) and children's acceptance of pseudoerrors.

In first aim, the results provide support to those obtained previously because children failed to differentiate between logical and conventional counting rules (LeFevre et al., 2006; Kamawar et al., 2010; Rodríguez et al., 2013; Escudero et al., 2015; Lago et al., 2016, 2019). Specifically, they rejected both compensation errors and pseudoerrors as examples of incorrect counting performance. Although compensation errors are non-routine trials, since presumably children have rarely seen counting strategies like those performed by the characters, children successfully rejected them. The same did not happen with pseudoerrors, which were also rejected by children, because they incorrectly assumed that the transgressed conventional rule was essential for correct counting. In fact, success rates of children were low and very close among the different age groups, underscoring the slow pace of development to improve this understanding during primary school.

The high success rate in compensation errors, with levels similar to those found with conventional errors where logical errors occurs one at a time and lead to incorrect cardinal values (e.g., Gelman and Meck, 1983; Briars and Siegler, 1984; LeFevre et al., 2006; Kamawar et al., 2010; Rodríguez et al., 2013), corroborate that the process of keeping track of the counted and uncounted items posed minimal performance demands for children. This can be observed in their justifications, where just the 14.1% of the participants detected only one of the two transgressions in compensation errors in more than one trial. Likewise, the analysis of the justifications given by the children showed that the vast majority of them could reproduce the breaches of conventional rules made by the characters in pseudoerrors (also in line with previous studies, such as Rodríguez et al., 2013; Escudero et al., 2015; Lago et al., 2016).

Several conclusions can be drawn from these findings. First, that the failures of the children when detecting non-routine trials are not related to memory problems, at least in the children of the age range here considered. Second, judgments in pseudoerror detection do not misrepresent understanding of children on the conventional counting rules. On the whole, children do not seem to be able to distinguish between acceptable and unacceptable counting in terms of logical and conventional rules, as evidenced in the rejection of correct non-standard counts. And lastly, children can be credited with a comprehension of logical rules but their grasp of conventional ones need to improve.

The second goal concerns a critical issue of failures of children to distinguish logical from conventional counting rules: their sensitivity to those failures. Expressed in other terms, do children demonstrate successful performance monitoring skills in a counting detection task? Consistent with the findings of many recent studies (Lyons and Ghetti, 2010; De Neys et al., 2014; Vo et al., 2014; Lubin et al., 2015), the data show that overconfidence is not an all-or-nothing phenomenon. The magnitude of overconfidence in young children with regard to their responses to the detection task differed between groups and tasks. The overall level of confidence was not too high; it was rather medium. Also, contrary to what might be expected, the bias scores of the kindergarten children were not significantly different from those obtained by older children (0.43 and 0.37, respectively). However, the first graders (0.52) were more overconfident than the second graders. This same pattern was evidenced in the absolute accuracy index, 5- and 7-year-olds were moderately accurate (0.47 in both cases), and the first graders (0.57) were slightly less accurate than them. And again with respect to the discrimination index, which indicated that the first graders were more confident about incorrect responses, whereas kindergartners and second graders were more confident about the correct responses. Thus, even the youngest participants exhibited moderate metacognitive monitoring skills.

As for the differences observed between the tasks, our participants were largely overconfident and inaccurate regarding their decisions about pseudoerrors (0.66 and 0.7, respectively) but showed low overestimation of their performance, and also greater accuracy (0.21 and 0.3, respectively), in relation to their judgments about compensation errors. Children's pseudoerror detection performance was as low as previously reported in several studies due to the power of conventional rules (Kamawar et al., 2010; Rodríguez et al., 2013), but confidence judgments were high. In sum, as expected, children did not seem to be sensitive to their incorrect performance when detecting counting pseudoerrors. In other words, they are unaware of their misconceptions about the nature of conventional counting rules.

A U-shaped curve was apparently observed across grade levels on the metacognitive measures. The performance of children was relatively good in kindergarten, became worse in the first grade, and improved again in the second grade. However, we consider it premature to assume that this response pattern is a developmental pattern of metacognitive abilities of children. On the one hand, to characterize such a developmental trend, many studies with a wide age-span and across routine and non-routine tasks are needed. On the other hand, as these metacognitive measures involve an appraisal of one's outcome in a task, they are linked to children's performance on detection counting tasks. Even though the success of the children in the counting tasks slowly improved as they grew older, most of our participants did not notice their failures, being highly confident about the accuracy of their responses. In fact, only the first graders performed significantly worse than the second graders on the counting detection tasks, which makes their metacognitive measures worse as well.

In our opinion, the reason behind the poor performance of the first grade children may lie in the fact that they experience the transition to formal schooling, during which they show some decline in performance due to the stricter criteria they employ when judging pseudoerrors. Typical mathematics instruction at this stage emphasizes arithmetic and rehearsal routines repeatedly, such as the use of the standard counting procedure as a means to solve addition and/or subtraction problems. More generally, this result could be understood as a symptom of the disconnection between the formal learning in the classroom and the informal learning activities. This disconnection does not allow children to integrate knowledge adequately, and much less when non-routine tasks are presented. In this case, for instance, children are often encouraged to use a single way of counting, emphasizing the rote procedure. In other words, the single way of counting promotes routine expertise (De Corte, 2012). The focus on the development of routine expertise fosters the ability to count accurately by following both logical and conventional rules. Adults consider this way of counting to be more controllable and less risky, so children rarely have the opportunity to practice alternative valid procedures. As proposed in the change resistance approach (Luchins and Luchins, 1950; McNeil and Alibali, 2005; McNeil, 2007), there could be some interference of these mechanically learned counting skills with the incorporation of new concepts and/or procedures as children progress through formal schooling approach.

Testimony of others is an undeniable source of learning (Corriveau and Harris, 2010; Pham and Buchsbaum, 2020). Some recent findings have identified several factors that affect the decisions of children about what information to support (Corriveau and Harris, 2010; Jaswal et al., 2010; Chan and Tardif, 2013; Lane et al., 2014; McGuigan and Burgess, 2017; Li et al., 2019). The third goal of the present study concerned a new factor, the influence of the argumentation of the testimony. Specifically, we examined the ability of the children to conform selectively to the testimony of a unanimous majority that justified or did not justify their claims.

Replicating previous research findings, from the same field of knowledge as the current study, children did not blindly accept counterintuitive testimonies of others about conventional rules of counting (Enesco et al., 2017; Lago et al., 2019). Regardless of their age and in accordance with the informational influence hypothesis, the participants conformed more often after hearing a justification of the unanimous testimonies (42.9% of the pseudoerrors) than after simply hearing the unanimous testimonies of the teachers (7.5% of the pseudoerrors), because they not only considered the content of the testimonies but also the justifications supporting them. This seems to suggest that conformity of children emerge from an informational rather than a normative motivation (Deutsch and Gerard, 1955). The explanations given by the children to argument their decisions to conform may offer support to this claim as well. The reasons of children to conform explicitly referred to information similar to the one unanimously reported by the teachers in 56.19% of the cases, opposite to 3.51% of the cases in which they alluded to the epistemic authority of the informants as the reason of their endorsing to the testimony. It is worth noticing that children did not merely parrot what they listened to the informants; they elaborated their judgments instead.

Although the nature of the transferred knowledge is the same in all these studies, since the content of the testimonies focused on logical and conventional rules of counting, there are some differences. For instance, contrary to Enesco et al. (2017) and Lago et al. (2019), the testimonies of the current study were exclusively unanimous and the teachers did not always provide reasoned justifications to explain their claims about the correctness of the pseudoerrors. The low overall probability of conformity of children could be attributed to their own erroneous counting knowledge that prevents them from endorsing counterintuitive claims. That is, the children were unwilling to endorse the testimonies of teachers about pseudoerrors as correct counting performances. In fact, only 56.9 and 12.2% of the children conformed, in at least one pseudoerror, when the teachers either provided a justification or not, respectively. We obtained a rate of conformity of 42.9% of the trials, a percentage that is consistent with those observed by Asch (1956), Haun and Tomasello (2011), and Lago et al. (2019). At least in the domain of number, which constitutes a naturalistic and ecologically valid context for exploring the influence of a majority testimony, children did not blindly follow the claims of an epistemically competent majority. They listened to the judgments and weighed up the arguments given by the teachers, showing a clear preference for testimonies with arguments to conform. Flynn et al. (2018) also observed that the conformity of the youngest children changed in different domains depending on whether they were exposed to unanimous agreement among four adults in a domain involving a conflict with their personal view or not involving such a conflict. Children did not conform blindly. They modulated their conformity in response to the domain-relevant information, conforming less when the domain was characterized by a conflict of the unanimous agreement of the adults with their personal view, than when the domain did not entail such a conflict.

The findings seem to lend support to the hypothesis that the conformity of children follows the same structure found in adult studies (several examples are collected by Haun et al., 2013). One such piece of evidence focuses on studies using Asch's (1956) paradigm that showed consistent conformity rates in children and adults. As is well-known, adults defer to a majority in about one-third of trials and, approximately, the conformity rates of the children ranged from 27 to 37% of trials in Haun and Tomasello's (2011) study, was 42.3% of trials in Lago et al. (2019), and 42.9% of trials in the present study. Apart from other considerations, these studies tested the compliance of children with the Asch's paradigm with methodological changes to make it child-friendly; the only one showing slightly lower conformity rates (ranging from 19 to 29% of trials) is that of Corriveau and Harris (2010), who nevertheless defend such parallelism between adults and children, beyond these small quantitative differences.

We also analyzed whether overconfidence in their own wrong responses would lessen the conformity of children. The confidence of children in their own answers did not condition their tendency to conform. The data indicated that the absolute accuracy index was not a predictor of the conformity of children in neither of the pseudoerrors. The magnitude of overconfidence was medium and was not an obstacle to accepting the testimony of teachers, especially when the teachers justified their claims. Probably because when the teachers articulated their arguments, the lack of logical consequences for violating conventional counting rules became evident. This performance pattern contrasts with that observed by De Neys et al. (2014) because 90.5% of their participants resisted the countersuggestion. None of the non-conservers, who doubted their erroneous responses, changed them after countersuggestion. This finding may be related to the fact that the countersuggestion offers no arguments, contains only the opposite response, and not to the fact that it comes from the children of the same age. As McGuigan and Burgess (2017) recently found, the age of the members of the majority relative to the observer influenced the tendency of the children to conform. Informants of the same age and older elicited conformity; younger informants did not elicit such a strong conformity. The findings are more in line with previous studies about the relationships between metacognitive abilities and academic tasks, such as Roebers et al. (2012), who observed that the influence of metacognitive skills in the school achievement of second graders (at the end of the study) was slighter in the context of mathematics than in the context of literacy.

The inconsistency between conformity and confidence judgments, where 89.1% of the children who conformed chose the two highest scores of the confidence scale, raises the question of whether it might be an indication that young children can only manifest states of knowledge or lack of knowledge, but not a more graded knowledge or uncertainty (Lyons and Ghetti, 2010, 2011). Future research in this issue is clearly needed due to the relevance of this field of knowledge to children's academic success in mathematics. Additionally, future research should address the study of factors that potentially explain individual differences in children's understanding of conventional counting rules, such as executive functions. Some authors (e.g., Van der Ven et al., 2012) have also noticed the convenience to investigate the relationships between executive functions and different specific mathematical skills. Finally, from a developmental perspective, it is also desirable to extend the age-range studied.

Despite these limitations, the current study has several educational implications, since a greater amount of time is invested at schools in order to teach children how to count. Teachers tend to model the standard procedure and children repeat that counting. Reflections about its meaning seem scarce in the school context, but merely mimicking the standard counting procedure may delay their grasping of a full understanding of counting (Paliwal and Baroody, 2020).

At this point, miscounts of children and explicit discussions of teachers on any kind of procedure (i.e., correct, incorrect, routine, and non-routine) is a promising educational approach that may foster conceptual development of children in this domain. Instead of being discarded, teachers should handle errors of children as sources of learning, for example, by exploring the logical consequences of different counting procedures (Freeman et al., 2000; Siegler, 2003; Loibl and Leuders, 2019). The explicit elaborations of the teachers on transgressions about logical and conventional counting rules (e.g., by prompting reflections of children about what is necessary—logical rules—and what is purely conventional) may help them not only to become aware of their misconceptions, but also to override them.

We also agree with Muldoon et al. (2003) (see also Freeman et al., 2000; Muldoon et al., 2007) in the need to reconceptualize counting errors. As expressed by Muldoon et al. (2007), children shift from disregarding miscounts as functionally irrelevant to recognize that they have logical consequences that can be corrected, not simply dismissed until a recount is done. To do this, children have to understand the relative value of conventional counting rules compared to the essential nature of logical rules. For instance, for a child to admit that an error of omission can be corrected by going back and counting the skipped element, she or he needs to understand that the violation of the spatial adjacency is irrelevant to an appropriate counting strategy. Conventional rules of counting could hinder counting development of children if they do not prioritize logical rules over conventional rules, or if they cannot apply the rules flexibly (Rodríguez et al., 2013).

## Data Availability Statement

The raw data supporting the conclusions of this article will be made available by the authors, without undue reservation.

## Ethics Statement

The studies involving human participants were reviewed and approved by the Deontological Commission of Psychology, Complutense University of Madrid. Written informed consent to participate in this study was provided by the participants' legal guardian/next of kin.

## Author Contributions

MOL, AE, and CD have equally contributed to the design of this work, data collection, its analysis, and interpretation of results. All authors contributed to the article and approved the submitted version.

## Conflict of Interest

The authors declare that the research was conducted in the absence of any commercial or financial relationships that could be construed as a potential conflict of interest.

## Footnotes

^1^The latest version of the software “Little House of Numbers” (Intellectual Property Number: M-002197/2012), used in previous studies.

^2^Formula for bias index: $\frac{1}{N}\overset{N}{\underset{i=1}{\sum }}\left(c_{i}-p_{i}\right)$, where *c* is the confidence rating and *p* is the real performance.

^3^Formula for absolute accuracy: $\frac{1}{N}\sum_{i=1}^{N}(c_{i}-p_{i}{)}^{2}$, where *c* is the confidence rating and *p* is the real performance.

^4^Formula for discrimination index: $\frac{1}{N}\left[\overset{N_{c}}{\underset{i=1}{\sum }}\left(c_{i\;correct}\right)-\overset{N_{i}}{\underset{i=1}{\sum }}\left(c_{i\;incorrect}\right)\right]$, where *c* is the confidence rating.

^5^We also conducted the regression model with bias as the metacognitive predictor to consider its influence. This did not alter the results, so we focused on absolute accuracy because it provides a straightforward interpretation of the precision of children's confidence judgments about pseudoerrors.
